# Emergence of a new lagovirus related to *Rabbit Haemorrhagic Disease Virus*

**DOI:** 10.1186/1297-9716-44-81

**Published:** 2013-09-08

**Authors:** Ghislaine Le Gall-Reculé, Antonio Lavazza, Stéphane Marchandeau, Stéphane Bertagnoli, Françoise Zwingelstein, Patrizia Cavadini, Nicola Martinelli, Guerino Lombardi, Jean-Luc Guérin, Evelyne Lemaitre, Anouk Decors, Samuel Boucher, Bernadette Le Normand, Lorenzo Capucci

**Affiliations:** 1 Anses, French Agency for Food, Environmental and Occupational Health & Safety, Ploufragan-Plouzané Laboratory, Avian and Rabbit Virology, Immunology and Parasitology Unit, BP 53, 22440 Ploufragan, France; 2 European University of Brittany, 5 Boulevard Laennec, Rennes 35000, France; 3 IZSLER, Istituto Zooprofilattico Sperimentale della Lombardia e dell’Emilia Romagna, 25124 Brescia, Italy; 4 ONCFS, National Hunting and Wildlife Agency, Department of Studies and Research, CNERA, CS 42355, 44323 Nantes Cedex 3, France; 5 INRA/ENVT, National Institute for the Agronomical Research/National Veterinary School of Toulouse, University of Toulouse, UMR1225, 31076 Toulouse, France; 6 ONCFS, National Hunting and Wildlife Agency, Department of Studies and Research, Unité Sanitaire de la Faune, BP 20, 78610 Le Perray-en-Yvelines, France; 7 Labovet Conseil, BP 539, 85505 Les Herbiers Cedex, France; 8 Clinique Vétérinaire Des Marches de Bretagne, 47 bd Leclerc, 35460 Saint-Brice-en-Cogles, France

## Abstract

Since summer 2010, numerous cases of *Rabbit Haemorrhagic Disease* (RHD) have been reported in north-western France both in rabbitries, affecting RHD-vaccinated rabbits, and in wild populations. We demonstrate that the aetiological agent was a lagovirus phylogenetically distinct from other lagoviruses and which presents a unique antigenic profile. Experimental results show that the disease differs from RHD in terms of disease duration, mortality rates, higher occurrence of subacute/chronic forms and that partial cross-protection occurs between RHDV and the new RHDV variant, designated RHDV2. These data support the hypothesis that RHDV2 is a new member of the *Lagovirus* genus. A molecular epidemiology study detected RHDV2 in France a few months before the first recorded cases and revealed that one year after its discovery it had spread throughout the country and had almost replaced RHDV strains. RHDV2 was detected in continental Italy in June 2011, then four months later in Sardinia.

## Introduction

*Rabbit Haemorrhagic Disease* (RHD) is a highly infectious and often fatal viral disease of the European rabbit *Oryctolagus cuniculus*. It was first described in China in 1984 [1] and is currently enzootic in wild populations in Europe but also in Australia and New Zealand where it was introduced as a biocontrol agent [2]. Periodic cases have been reported in domestic rabbits in other parts of the world including the Americas. When it emerged, RHD strongly affected wild populations and was responsible for great economic losses in the rabbit industry [3,4]. The development of efficient commercial vaccines that totally protect against the disease has enabled its control in rabbitries [5].

Susceptibility to the disease begins in the 5-6^th^ week of life of rabbits and steadily increases up to the 8-9^th^ week when they become fully susceptible. RHD is mainly characterised by a peracute/acute course with high mortality, up to 80–100%, in 48 to 96 h [6]. During an outbreak, 5 to 10% of rabbits may show subacute/chronic evolution of the disease. They can die 1 or 2 weeks later, probably due to liver dysfunction, or recover and seroconvert with very high antibody titres [7]. Typical post-mortem lesions include hepatic necrosis and splenomegaly. In a variety of organs and tissues, congestion and haemorrhages resulting from a disseminated intravascular coagulation syndrome are common. The trachea is often hyperaemic and contains frothy, bloodstained mucus [6].

The aetiological agent of the disease, the *Rabbit Haemorrhagic Disease Virus* (RHDV), is a non-enveloped single-stranded positive-sense RNA virus [8-11] belonging to the genus *Lagovirus* of the family *Caliciviridae*[12]. Only one serotype is known, including the antigenic variant named RHDVa, which was identified simultaneously in 1996–1997 in Germany and Italy [13,14], before spreading throughout the world. Although RHDV is an RNA virus, it shows low genetic variability [15-18]. Nevertheless, different studies have pointed out that RHDV is distributed into several phylogenetically distinct groups. French RHDV isolates have been assigned into 5 genetic groups (G1 to G5) following a temporal distribution [16]. More recently, RHDV was shown to cluster into 3 major groups, one of which includes the French genetic groups G3, G4 and G5 [18,19]. RHDVa forms a distinct genetic group (G6 according to [16], Group 1 or Clade D according to [18] or [19]) but cross-protection is almost complete between RHDV and RHDVa.

Since the 1990’s, different non-pathogenic or moderately pathogenic rabbit lagoviruses genetically related to but relatively distant from RHDV have been described, highlighting the extent of diversity within the lagoviruses [20-25]. The first non-pathogenic lagovirus known as the rabbit calicivirus (RCV) was identified in the small intestines of healthy domestic rabbits in Italy in 1996 [20]. Afterwards, several distinct non-pathogenic lagoviruses were identified in Europe [22,23,25] and Australia [21] in domestic or/and wild rabbits. Experimental studies have proven the existence of a gradient of cross-protection between these non-pathogenic strains and RHDV, from non-protective (strain 06–11) [25] and partially protective (RCV-A1) [26] to fully protective (RCV) [20]. Recently, a possible moderately pathogenic rabbit lagovirus (Michigan rabbit calicivirus, MRCV) was characterised following mortalities in a rabbitry in 2001 in the USA [24]. Its pathogenicity remains doubtful since it was isolated in dead rabbits showing clinical signs suggesting RHD but for which experimental infections failed to reproduce the disease.

In August 2010, one atypical case of RHD was reported in a rabbitry in northern France in which 25% of RHDV-vaccinated does died, as well as numerous non-vaccinated fattening rabbits [27]. A few similar cases were then reported, but their number markedly increased in north-western France as of October 2010. During the same period, several outbreaks were reported in wild rabbit populations in the same area. Macroscopic and microscopic examinations of organs revealed typical RHD lesions [27]. Samples collected in mid-October in north-western France from domestic and wild rabbits revealed the presence of a virus genetically related to but distant from RHDV and RHDVa isolates. Its phylogenetic relationships with known pathogenic and non-pathogenic rabbit lagoviruses established from a partial nucleotide sequence of the capsid protein VP60 (354 bp long) suggested that it was a new genetic group [27]. The new RHDV variant, hereafter referred to as RHDV2, was subsequently identified for the first time in Italy in one industrial farm in the Udine province (north-eastern Italy) in June 2011 [28].

In this work, the phylogenetic relationships of RHDV2 to other rabbit lagoviruses were confirmed by describing the complete sequence of the capsid protein gene. Its antigenic properties were determined and its pathogenicity was investigated in experimental studies. In addition, a molecular epidemiological survey of RHDV isolates collected in France between 2009 and 2011 was conducted to determine whether RHDV2 was present before its first detection and to describe its spread throughout France. The presence of cases of RHDV2 in Italy was also sought.

## Materials and methods

### Biological samples

Dead rabbits were collected from rabbitries affected by RHD to characterise the viruses involved in these outbreaks. New Zealand White RHDV-vaccinated does (case 10–05), non-vaccinated fattening rabbits (cases 10–07, 10–28 and 10–32), and young rabbits just before weaning (4-week-olds, case 10–08) were collected in 5 industrial rabbitries located in north-western France between September and November 2010. In addition, samples were collected in two epidemiologically related farms in the Udine province (north-eastern Italy) in June and July 2011 (case Ud11). In all cases, post-mortem examinations of dead rabbits revealed typical RHD lesions.

A molecular epidemiological study was carried out to monitor the spread of RHDV2 in France. We performed RT-PCR and genotyping analyses to describe the isolates involved in RHD outbreaks starting in 2009. For this purpose we analysed liver specimens of 191 wild rabbits whose deaths were attributed to RHD, collected throughout France between January 2009 and December 2011 by the SAGIR network (French Wildlife Health Surveillance Network, [29]). Some of the analysed samples belong to the same epizootic. The presence in these samples of RHDV had previously been confirmed by the Anjou Laboratory (Angers, France) using an RHDV ELISA test [30]. In addition, we analysed liver samples of domestic rabbits that died from RHD: one archived RHD case (case 10–01) that occurred in April 2010 in a rabbitry in north-western France and 70 rabbits reared in 63 industrial rabbitries that died of RHD between October 2010 and December 2011.

Regarding the presence of RHDV2 in Italy, information was obtained as part of the existing epidemiological surveillance plan for RHD, further implemented after the identification of the first case of RHDV2 in June 2011. Thus, 74 samples were collected between November 2009 and October 2011 in domestic and wild rabbits from different parts of Italy. Post-mortem analyses of liver samples were carried out using a diagnostic ELISA test [5] to determine whether RHDV was the cause of the observed mortalities. The antigenic profile of each RHDV isolate was then identified with a sandwich ELISA (described in the chapter below) in order to determine its group (RHDV, RHDVa or RHDV2). In addition, a few rabbit samples collected between October 2011 and early 2012 in Sardinia and the Trentino region (northern Italy) where RHDV outbreaks occurred were characterised.

### Antigenic characterisation

The antigenic profile of the RHDV2 strains 10–28, 10–32 and Ud11, the RHDV reference strain Bs89 (X87607), the RHDVa reference strain Pv97 (EU250330), and the EBHSV reference strain Bs89 (X98002) were compared using sandwich ELISA with a panel of 26 monoclonal antibodies (MAbs) produced for RHDV (16 MAbs) or EBHSV (10 MAbs). On the basis of their reactivity, they were divided into 4 subsets: i) 2A10, 1H3 and 1H8 are specific to RHDV and recognise at least two independent epitopes [31], ii) 3B12, 3D4, 3D6, 5D11 and 2E1 are specific to RHDVa and recognise at least 3 independent epitopes [32], iii) 2B4, 2G3, 1F10, 6H6, 3H2, 6D6, 6 F9 and 3H6 react with both RHDV and RHDVa and recognise at least 6 independent epitopes. The last 3MAbs (6D6, 6F9 and 3H6), although produced for RHDV, also cross-react with EBHSV indicating that the corresponding epitopes are common to these lagoviruses [31], iv) 2B2, 1C5, 3D6, 4E3, 1F8, 5 F5, 1G8, 1H1, 1H12 and 4H4 are specific to EBHSV.

For the sandwich ELISA, convalescent anti-RHDV rabbit sera were adsorbed to a Nunc Maxisorp ELISA plate at a dilution of 1/5000 in standard carbonate buffer. Ten percent liver homogenate RHDV positive was pre-titrated in ELISA [5] and used at a dilution that gave an OD_492_ value in the range of 1.2-1.5. MAbs were used in the range of 20–200 ng/mL depending on the reactivity with the homologous virus. Finally, a rabbit IgG anti mouse IgG labelled with HRP was used to detect binding of the MAbs to the viruses. In order to control the reactivity of MAbs anti-EBHSV, a sandwich ELISA using a hare serum anti-EBHSV adsorbed to the solid phase and the reference strain EBHSV BS89 were additionally employed.

### Haemagglutination (HA) test

An HA test was performed for assessing the haemagglutinating properties of RHDV2 towards human type “O” erythrocytes in comparison with reference RHDV and RHDVa strains, and for estimating the viral load of the inocula used in the experimental studies. Samples containing the strains 10–28, 10–32 and Ud11 were tested using the classical protocol with human group “O” red blood cells (RBC) [5]. Briefly, the HA test was performed at 4 °C with 10% liver homogenate in PBS pH 7.4 and washed RBC diluted to 1% in PBS pH 7.2.

### RT-PCR and sequence analysis

The VP60 gene sequences of the 7 RHDV2 strains isolated from dead rabbits collected from rabbitries in France and Italy (cases 10–01, 10–05, 10–07, 10–08, 10–28, 10–32, Ud11) were determined. RNA was extracted from 100 μL of liver exudate using the RNeasy Mini kit (QIAGEN) or from 10 μL of liver homogenised in PBS (100 mg/mL) with TRIzol® Reagent (Invitrogen). They were reverse transcribed using oligo-dT (Invitrogen) as a primer and Superscript™ II Reverse Transcriptase (Invitrogen). The capsid protein VP60 gene sequences of the French viruses were obtained following full-length gene PCR amplification using the primers “12U” (5’-GTCGTCTCGGTAGTACCTG-3’) and “15 L” (5’-ATCAAGCACTGGACTCGCC-3’). The amplified PCR products (2116 bp) were visualised by electrophoresis on agarose gel and purified prior to sequencing. The DNA sequence was determined twice in both directions by the dye terminator method (Applera Applied Biosystems) using the PCR primers “12U” and “15 L” as well as several primers designed in the inner region of the DNA template (primer sequences available upon request). The Italian Ud11 VP60 gene sequence was obtained following 3 overlapping PCR (PCR1: 5225-F 5’-GCTACGATGCTGCTAGGAAGATACT-3’ and RHDV-R 5’-AACCCTCCAGGTACTGGTTG-3; PCR2: RHDV-F 5’-CCTGTTACCATCACCATGCC -3’ and 6694-R 5’-CGAACATGATGGGTGTGTTC-3’; PCR3: 9510-F 5’-ACATACACACCTCAACCAGA-3’ and 109-R 5’-CGCCGGCGCCTGCAAGTCCCAATCC-3’). The amplified templates were purified and sequenced as mentioned above using the PCR primers. The deduced amino acid sequences of the VP60 genes were obtained using the “EMBOSS Transeq” software available on the EMBL-EBI website. Nucleotide or amino acid sequence alignments were generated by the CLUSTAL W method [33] using the NPS@) software [34] from the PBIL website.

For the molecular epidemiological survey of French RHDV isolates, we used the PCR primers “14U1” (5’-GAATGTGCTTGAGTTYTGGTA) and “RVP60-L1” (5’-CAAGTCCCAGTCCRATRAA) to amplify 794 pb located in the C-terminal of the gene encoding VP60. These amplified templates were subsequently sequenced using the PCR primers. In order to genotype the isolates, the sequences were aligned and compared with lagovirus sequences representative of the different RHDV genetic groups [16,18,19], the non-pathogenic lagoviruses, and RHDV2.

### Phylogenetic analyses

Phylogenetic relationships were inferred using the 7 French and Italian RHDV2 VP60 gene sequences obtained from this study and the complete rabbit lagovirus VP60 gene sequences available in databases. The RHDV strain Hartmannsdorf (Y15426) for which evidence of recombination in the capsid region of the genome was shown [18,35] was excluded as well as identical sequences corresponding to the same viral strain but with different accession numbers. Thus, 46 RHDV, 34 RHDVa, the non-pathogenic RCV and 06–11 strains, 36 non-pathogenic RCV-A1 isolates and the possibly moderately pathogenic MRCV were analyzed along with the 7 RHDV2. An additional file shows the sequence names and their accession numbers (see Additional file 1). The sequence of the French reference *European Brown Hare Syndrome Virus* (EBHSV) strain EBHSV-GD (Z69620) was used as an outgroup to root the trees. Different phylogenetic analyses were conducted using MEGA software version 5 [36]. Phenetic (Neighbor-joining (NJ) and Minimum Evolution (ME) methods) and Maximum Likelihood (ML) analyses were implemented with the pairwise deletion option and based on the Kimura 2-parameter model including transition and translation substitutions. For the cladistic analysis, the Maximum Parsimony (MP) method was used and the pairwise deletion option was selected. For all the methods, the codon positions included were 1st + 2nd + 3rd. For the ME, ML and MP methods, the search for the trees was implemented with the close-neighbor-interchange algorithm. Reliability of the trees was assessed by bootstrap with 1000 replicates, except for the ML method (500 replicates). Pairwise nucleotide distance comparisons based on the p-distance model were conducted using MEGA5.

### Recombination analysis

To determine whether recombination events occurred within the VP60 gene of RHDV2, the capsid gene sequences available in databases and the RHDV2 sequence were screened using RDP4 Beta 4.16 software [37].

### Experimental infections

In order to study the pathogenicity of RHDV2 and the induced pathological lesions, several experimental studies were performed under negative pressure in BSL2 (France) and BL3 (Italy) experimental facilities. In France, the animal experiments were carried out in accordance with the guidelines of the European Community Council on Animal Care (86/609/CEE) and approved by the ethical committee of the Veterinary College Scientific Council. In Italy, they were performed in compliance with the provisions of national and European laws (DM 2894/95 and DLgs 116/92, receipt of 86/609/CEE).

RHDV seronegative rabbits aged ten weeks and older were used. Their seronegativity was checked with a VP60-RHDV ELISA based on the detection of a baculovirus expressed capsid protein, as described in [25] or with a competition ELISA (cELISA) used for the serology of RHDV classical strains [38]. The rabbits were New Zealand rabbits coming from commercial suppliers and unselected rabbits coming from a rural unit. Depending on the experimental batch, the rabbits were inoculated by the intramuscular, oral, or intravenous route with 4 different inocula made up of supernatant (1 mL) of homogenised dead rabbit liver samples collected from 3 French rabbitries (strains 10–07, 10–28, and 10–32) and from the Italian farm (strain Ud11). The viral load of the different livers used for the infections was estimated using the HA test and gave titres of between 1/5120 and 1/10 240. The inocula were prepared according to approximately the same protocol (1/10-1/20 dilution of the supernatant of at least 10% w/v homogenised liver in PBS). In two studies (A and G), one group of rabbits was challenged with a standardised inoculation of 10^3^ LD_50_ of RHDV reference strain (V/RHD/4) isolated in 1988 in France [39] as a control. In addition, in studies F, I, and L, 18 seropositive survivor rabbits were challenged one month later with approximately 2.10^3^ LD_50_ of the Italian reference RHDV strain BS89 [40] to estimate the level of protection induced by the RHDV2 strain.

Daily observations for morbidity (asthenia, anorexia, respiratory and nervous signs) and mortality were performed. Dead animals, as well as animals surviving at the end of the experimental study and which were anaesthetised before being killed, were examined for macroscopic lesions. Liver samples were collected to confirm the cause of death by RT-PCR analyses. Except for the A, B, and G experiments, blood samples were collected from survivor rabbits two weeks post inoculation to check whether the rabbits seroconverted. Antibody titres were measured with the previously mentioned cELISA.

### Statistical analyses

The effects of viral strains on mortality rates were studied using χ^2^ tests.

### Nucleotide sequence accession numbers

Nucleotide sequences of RHDV2 strains are available in databases under the EMBL/GenBank accession numbers FR819781 (10–05), HE800529 to HE800532 (10–01, 10–07, 10–28 and 10–32, respectively), HE819400 (10–08) and JQ929052 (Ud11).

## Results

### Sequence analysis of the capsid protein

The 7 RHDV2 VP60 gene sequences are 1740 nucleotides long (579 amino acids long) and are closely related to each other (1.3% nucleotide difference on average). The average nucleotide identity between RHDV2 and RHDV-RHDVa is 82.4%. The RHDV2 share 82% identity on average with RCV, 06–11 and MRCV strains, and 80.6% identity with RCV-A1 isolates. Nucleotide identity is 70.4% with EBHSV, showing that RHDV2 is closer to rabbit lagoviruses. Amino acid similarity between the 7 RHDV2 strains is 99.3%, whereas average similarity is about 89.2% with the RHDV-RHDVa, RCV, 06–11, and MRCV strains, and 87.4% with RCV-A1 isolates. Amino acid similarity is 76.7% with EBHSV.

**Figure 1:**
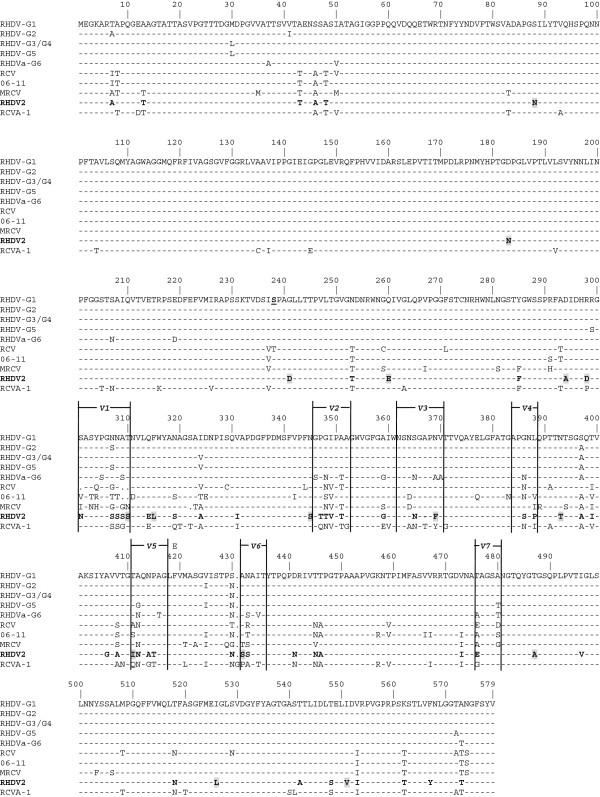
**Consensus amino acid sequence alignment of the VP60 gene from RHDV2 and other lagoviruses.** The aligned lagovirus sequences correspond to the consensus sequences of i) pathogenic RHDV and RHDVa sequences (47 sequences) belonging to the different genetic groups (G1 to G6) identified by Le Gall-Reculé et al. [16], ii) RCV-A1 sequences (36 sequences). Three other rabbit lagovirus sequences were aligned and correspond to the non-pathogenic RCV (X96868), the non-pathogenic 06–11 (AM268419) and the moderately pathogenic MRCV (GQ166866) strains. Residues differing from the first sequence are shown and a dot corresponds to a deletion. The hypervariable regions V1 to V7 according to Wang et al. [43] are indicated at the top of the alignment. The amino acid S238 (in bold and underlined in the first sequence) corresponds to the beginning of the protrusion domain of the VP60 protein [43]. The RHDV2 mutated amino acids shared by no other rabbit lagovirus are highlighted in grey.

The consensus sequence of the 7 RHDV2 deduced protein sequences was aligned with the consensus sequences of the RHDV sequences available in databases and belonging to the different genetic groups (G1 to G6) identified by Le Gall-Reculé et al. [16]. The genetic group G5 corresponds to the main genetic group that was circulating in France until the emergence of RHDV2. The MRCV, RCV, and 06–11 sequences, and the consensus sequence of RCV-A1 sequences were also included (Figure 1). Most of the substitutions are located in the most variable part of the capsid protein, the C-terminal region, which constitutes the protrusion (P) domain of the particle [41-43] and contains the main antigenic regions [31]. Twenty-five amino acid substitutions are only shared with some non-pathogenic viruses, sometimes including the MRCV, and 16 substitutions are shared by no rabbit lagoviruses (Figure 1). When we compared the RHDV2 sequence within the 7 regions of the P domain that show the highest degree of genetic variation (regions V1 to V7 [43]), similarities decreased to 65.3%, 63.2%, 60%, 59.2% and 53%, between RCV-A1, RCV, RHDV, MRCV and 06–11, respectively (Figure 1). These data emphasised the differences between the viruses.

The analyses failed to detect any recombination event with another lagovirus within the VP60 gene.

### Genetic relationships

**Figure 2:**
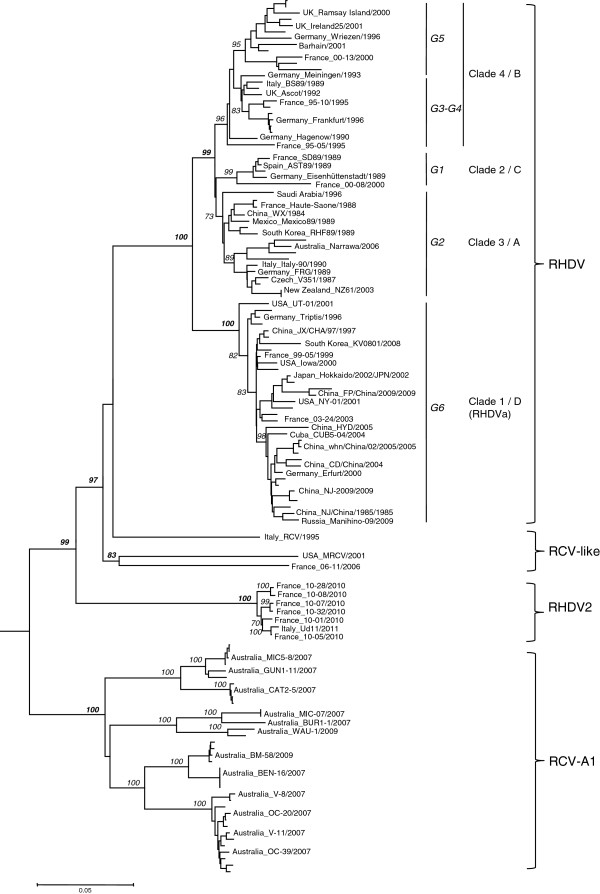
**Phylogenetic tree derived for VP60 gene nucleotide sequences of 127 rabbit lagoviruses including 7 RHDV2.** The tree was obtained using the Neighbor-Joining method and was drawn to a scale of nucleotide substitutions per site. The percentages greater than 70% of replicate trees in which the associated taxa clustered together in the bootstrap test (1000 replicates) are given in italics before each major branch node. *The European brown hare syndrome virus* (EBHSV) strain GD (Z69620) was used as an outgroup to root the tree. The names of some representative strains from different countries are shown. For RHDV, the genetic groups G1 to G6 according to Le Gall-Reculé et al. [16] and clade 1 to 4, or A to D, according to Kerr et al. [18] or to Kinnear et al. [19], respectively, are annotated.

Phylogenetic analyses based on the VP60 gene nucleotide sequences of the 7 studied RHDV2 strains and the available rabbit lagoviruses in the databases gave similar results irrespective of the method used. We show the tree generated by the Neighbor-Joining method (Figure 2). The results revealed that the rabbit lagoviruses were clustered into four highly supported phylogenetic groups, (i) a first group including the pathogenic RHDV and RHDVa isolates, (ii) a second group including the MRCV, 06–11, and Ashington strains, (iii) a third group including the RHDV2 strains, and (iv) a fourth group including the RCV-A1 isolates. The RCV forms a separate branch without a significant bootstrap value but regardless of the phylogenetic analysis used, it always clusters into the large group consisting of the first and the second genetic groups. The new phylogenetic group formed by RHDV2 was more closely related to RHDV and RCV-like viruses than to the independent RCV-A1 genetic group.

### Antigenic characterisation and HA properties

Using ELISA, no specific MAb belonging to the subsets i and ii reacted with RHDV2; nor did MAbs 2B4 and 2G3 of the subset iii (Table 1). In addition, MAbs 1F10 and 6H6, belonging to the cross-reactive subset iii, showed a consistent decrease in reactivity with RHDV2 (about 85% and 50%, respectively). As a consequence, the reactivity with the available MAbs might suggest that RHDV2 is antigenically close to EBHSV. However, the 10 specific EBHSV MAbs did not react with RHDV2, or with RHDV or RHDVa. These results show that, in agreement with the above genetic data on the 7 regions that show the highest degree of genetic variation, the antigenic surface of RHDV2 was very different from that of both RHDV and RHDVa. The finding that most of the anti-RHDV MAbs not recognising RHDV2 do not recognise EBHSV either, suggests that the degree of antigenic difference between RHDV2 and the other RHDV was similar to that between EBHSV and the other RHDV.

**Table 1:** Antigenic characterisation of RHDV2 by ELISA using specific MAbs for RHDV and EBHSV reference strains.

**MAb**		**RHDV**	**RHDVa**	**EBHSV**	**RHDV2**
i)	2A10	100	0	0	0
	1H3	100	0	0	0
	1H8	100	0	0	0
ii)	3B12	0	100	0	0
	3D4	0	100	0	0
	3D6	0	100	0	0
	5D11	0	100	0	0
	2E1	0	100	0	0
iii)	2B4	100	100	0	0
	2G3	100	100	0	0
	1F10	100	100	0	15
	6H6	100	100	0	50
	3H2	100	100	0	100
	6D6	100	100	100	100
	2F9	100	100	100	100
	3H6	100	100	100	100
iv)	2B2	0	0	100	0
	1C5	0	0	100	0
	3D6	0	0	100	0
	4E3	0	0	100	0
	1F8	0	0	100	0
	5F5	0	0	100	0
	1G8	0	0	100	0
	1H1	0	0	100	0
	1H12	0	0	100	0
	4H4	0	0	100	0

On the basis of their reactivity, the 26 monoclonal antibodies (MAb) were divided into 4 subsets. The reactivity of each MAb is given in percentage. Thus, 100 corresponds to a reactivity of 100% whereas 0 corresponds to an absence of reactivity.

The 3 RHDV2 tested (10–28, 10–32 and Ud11) resulted in positive HA tests with titres ranging from 1/1280 to 1/20 480 according to the livers tested, i.e. similar to those usually found with most of the RHDV or RHDVa isolates. These results show that RHDV2 agglutinates human RBC of type “O” efficiently and confirmed that the HA test, which constitutes a routine diagnostic method still used in some veterinarian laboratories, can identify the presence of RHDV2 in infected samples.

### Experimental infections

The different experimental studies performed to determine the pathogenicity of RHDV2 confirmed its virulence regardless of the inoculation route (Table 2). Mortalities occurred later and over a longer period than with both classical RHDV and RHDVa: 3–9 days post-inoculation and lasting 5 days, instead of 2–6 days post-inoculation and lasting 3–4 days as generally observed with classical RHD. A few animals, especially in experiments that showed a higher level of mortality, presented an acute course of the disease and died within 3–4 days. Rabbits that developed a subacute/chronic course were more frequent than for classical RHD. Thus, the observed macroscopic lesions were typical of RHD but with a more frequent occurrence of the severe liver degeneration and discoloration, splenomegaly and jaundice that characterise the subacute/chronic form of RHD [6]. Rabbits that survived the infection did not show any clinical signs and were fed regularly.

**Table 2:** Mortality results following experimental challenges with 4 RHDV2 or 1 RHDV reference strains.

**Study**	**Rabbit origin**	**Administration route**	**Inoculum strain**	**Mortality**	**Seropositivity of the survivors (doubtful)**^**2**^	**Mortality 2nd challenge RHDV BS89**
**A**^**1**^	NZ	IM	10-07	1/5	/	
			V/RHD/4	3/5	/	
**B**^**1**^	NZ	oral	10-28	6/8	/	
**C**	NZ	IM	10-28	1/2	1/1	
		oral	10-28	2/2	/	
**D**	NZ	oral	10-28	3/9	6/6	
**E**	NZ	oral	10-28	0/5	5/5	
**F**	NZ	oral	Ud11	1/5	4/4	0/4^3^
**G**^**1**^	NZ	oral	10-32	0/8	/	
			V/RHD/4	8/8	/	
**H**	NZ	oral	10-32	0/3	3/3	
**I**	NZ	IV	10-32	0/4	4/4	0/1^3^
		oral	10-32	1/6	5/5	2/4^3^
**J**	NZ	oral	10-32	2/5	3/3	
**K**	NZ	oral	10-32	0/5	4(1)/5	
**L**	NZ	oral	10-32	0/5	5/5	0/4^3^
	Rural	oral	10-32	0/5	5/5	1/5

The experimental challenges were performed with RHDV2 strains 10–07, 10–28, 10–32, and Ud11, or with the French RHDV reference strain V/RHD/4, using different inoculation routes. For experiments F, I, and L, mortality results following a second challenge with the Italian RHDV reference strain BS89 of the survivor rabbits are shown.

*NZ* New Zealand, *IM* intramuscularly route, *IV* intravenous route.

^1^no serological data.

^2^doubtful because just above the threshold.

^3^the 2nd challenge was performed on a part of the survivor rabbits.

The serological analyses showed that the survivors were seropositive, demonstrating that all the rabbits had been successfully infected. However, the observed mortality rates were lower than with classical RHD and seemed highly variable (Table 2). The similarity between RHDV2 infected livers and RHDV or RHDVa infected livers with regard to HA titres showed that there was a similar amount of virus in the livers of dead rabbits, regardless of the virus. In addition, by using an almost identical protocol to prepare the different RHDV2 inocula, we may assume that we used about the same viral dose for each viral infection. Thus, the difference in mortality between the RHDV2 and RHDV-RHDVa viruses is most likely not due to a difference in the amount of viral particles inoculated. Most experiments were carried out on New Zealand rabbits inoculated by the oral route with the 10–28 or 10–32 strains (Table 2), which enable to test a strain on the mortality rates avoiding possible confounding factors. Statistical analyses on the pooled data for each strain showed a clear strain effect on the observed mortality rates (χ^2^ = 9.72, df = 1, *p* = 0.002) confirming that strain 10–28 caused a significantly higher mortality rate (46%, *n* = 24) than did strain 10–32 (9%, *n* = 32). The strain effect likely explains most of the observed variability in mortality rates in our experimental trials.

A second challenge with the reference RHDV BS89 strain was carried out on 18 seropositive rabbits that had survived an RHDV2 challenge (4 survivors of the Ud11 challenge in study F and 14 survivors of the 10–32 challenge in studies I and L; Table 2). Three of the rabbits died and the presence of the challenge viral strain in livers was confirmed. Thus, partial protection against RHDV was induced by anti-RHDV2 antibodies.

### Epidemiological study

**Figure 3:**
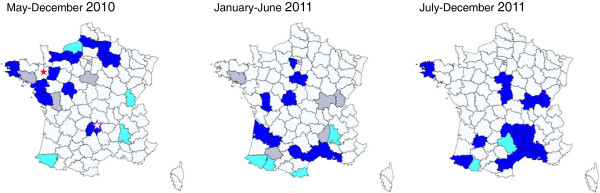
**Spatial distribution of RHD outbreaks in wild rabbit populations in France in 2010 and 2011.** Data are pooled by *département*. The color indicates the RHDV strains involved in these outbreaks. Light blue: classic RHDV; dark blue: RHDV2; squared: classic RHDV and RHDV2; white: no data. Red star: first detected outbreak of RHDV2 in a rabbitry (April 2010); white star: first detected case of RHDV2 in a wild population (May 2010).

The retrospective study carried out on RHDV isolates collected in France since January 2009 shows that the first occurrences of RHDV2 were detected in April 2010 in a rabbitry in western France (case 10–01) and in May 2010 in a wild rabbit in the centre of France. In wild populations from May to December 2010, i.e. after the virus detection, RHDV2 was responsible for 74% (25/34) of the recorded RHD epizootics, mainly located in north-western France (Figure 3). In 2011, RHDV2 was responsible for 73% (29/40) of the recorded epizootics between January and June, and for 95% (41/43) of the recorded epizootics between July and December. It was detected in southern France as early as February 2011, showing the large-scale spread of the virus (Figure 3). In domestic rabbits, RHDV2 was responsible for 93% of the recorded epizootics between October 2010 and December 2011 (54/58). In addition, we confirmed the disease in recently RHDV-vaccinated rabbits (3–4 months earlier) and the specificity of the clinical signs (subacute/chronic forms of RHD, higher mortality rates in 4-week-old rabbits).

In Italy, the antigenic profile of the 74 RHDV isolates identified between November 2009 and October 2011 showed that RHDV2 was involved in only two outbreaks that occurred in the Friuli region (Udine) in June and July 2011. These cases were epidemiologically linked since the rabbitries belong to the same owner. Since these initial cases, RHDV2 has been identified in two more regions. In Sardinia, at least 4 distinct cases were reported in October-December 2011, in both domestic and wild rabbits in different locations on the island (A. Puggioni, personal communications). In the Trentino region, an outbreak involving an open-space rural farm and the nearby wild populations was recorded in January-February 2012 (D. Dellamaria, personal communications). Therefore, as opposed to France, most of these RHDV cases were due to classical RHDV and RHDVa strains, and were found mainly in small rural units.

## Discussion

In 2010, we identified in both wild and commercially bred rabbits a pathogenic lagovirus which differs from RHDV in terms of phylogenetic position, antigenic profile and pathogenicity.

According to Kinnear and Linde [19], full-length capsid protein gene sequences provide a sufficient number of sequences and information per sequence to enable robust phylogenetic inference. Thus, the genetic relationships determined in this study on the complete capsid gene sequences confirm the results previously observed on a 354 bp-long fragment of the VP60 gene [27] and show that RHDV2 is phylogenetically distinct from all previously described members of the genus *Lagovirus* and forms a new genetic group. RHDV2 is less virulent than the previous described RHDV and RHDVa strains. In addition, the clinical characteristics of the induced disease are rather different from those described in “classical” RHD, notably in terms of disease duration, mortality rates and the occurrence of subacute/chronic forms, which are more frequent. The limited protection conferred by commercial vaccines as well as the mortality observed in convalescent RHDV2 rabbits when challenged with classical RHDV, show that cross-protection between RHDV and RHDV2 is partial. This partial cross-protection is consistent with the antigenic characteristics of RHDV2, which differ from RHDV and RHDVa. All these results suggest that RHDV2 can be considered to be a new member of the genus *Lagovirus*. We therefore propose to name this lagovirus RHDV2 to distinguish it from the original RHDV and RHDVa strains and to underline its membership in a second distinct lineage of pathogenic rabbit lagoviruses.

The observed differences in virulence between RHDV and RHDV2, and also in virulence among RHDV2, offer new insights into poorly documented host-virus interaction mechanisms in lagoviruses. The differences in pathogenicity could be related to the specificity of the virus for histo-blood group antigens (HBGA) involved in rabbit sensitivity to RHDV [44]. Another hypothesis is that small genetic divergences on specific sites may be responsible for the observed gradient of virulence between strains. The antigenic profile of RHDV2 indicates clear differences with RHDV and RHDVa in the viral surface where neutralizing epitopes are located. Some substitutions in the capsid gene specific to RHDV2 may explain these antigenic and pathogenic properties of RHDV2. Indeed, RHDV2 shows genetic variation within several regions of the capsid sequence that may contribute to the binding specificities of HBGA and that may be involved in RHDV antigenicity [43].

The epidemiological survey shows that the first documented cases of RHD caused by RHDV2 occurred in France in April and May 2010 in a rabbitry and in a wild population, respectively. This emergence remained undetected until the arrival of the first cases affecting vaccinated farmed rabbits in August 2010. RHDV2 then quickly spread throughout France, from the north-west to the south, and recently, after having crossed geographical barriers (the Alps and Pyrenees), also spread to two adjacent countries: Spain (May 2011) as confirmed by the phylogenetic analysis of two sequences available in databases (JQ 627641 [45] and JX133161 [46]) (data not shown), and Italy (June 2011). The rapid spread of the new virus was quite spectacular and can be partly explained by the imperfect protection of wild rabbits conferred by RHDV against RHDV2. Our data also show that RHDV2 has almost completely replaced classic RHDV isolates in France in both wild and domestic populations. This trend is a major concern for the rabbit industry since current commercial vaccines, which were developed for classic RHDV, have limited efficacy against RHDV2. The moderate virulence of RHDV2 is probably a selective advantage and may explain its ability to replace highly pathogenic RHDV in wild populations in France. This observation had been predicted by Fouchet et al. [47] using a model system which showed that moderately virulent RHDV-related strains invaded fragmented rabbit populations such as wild rabbit populations, with greater efficacy than highly virulent or non-pathogenic strains.

On the contrary to what happened in France, RHDV2 did not spread within continental Italy. Few cases of RHDV2 were reported between mid 2011 and early 2012, and most RHD outbreaks were caused by classical RHDV or RHDVa strains, mainly in small rural units. As a consequence the replacement of circulating RHDV or RHDVa isolates has not been observed. On the contrary in Sardinia, the observations were similar to those made in France since the rapid spread of RHDV2 was reported, with several cases detected in a very short period of time throughout the island. In most parts of France as in Sardinia, wild rabbits are widespread and present throughout the entire country, whereas in continental Italy wild rabbit populations are sparse and patchily distributed. This suggests that wild populations may exert strong selection pressure on the virus since the selective advantage of RHDV2 is only expressed where wild populations of rabbits are present.

The long distance between successive recorded cases from northern Italy to Sardinia, and also in France between the first detected case in a rabbitry in North-Western France and the second one in a wild population in central France, poses some epidemiological issues regarding the sources and routes of spread of the virus. The diffusion of the virus does not seem to be linked only to commercial trade since after the Udine outbreak no secondary outbreaks were detected in Italian industrial farms. An exhaustive epidemiological survey would be necessary to explain these different observations.

The emergence of RHDV2 raises the question of the origin of this new lagovirus. The molecular epidemiological investigations carried out regularly in France since the emergence of RHDV [14,48] and the last one performed on samples collected in 2008 (G. Le Gall-Reculé, unpublished data), did not detect the arrival of such a genetic shift. The two possible causes of emergence of RHDV2 are either the evolution of a pre-existing non-pathogenic virus or a species jump from a reservoir host species. Molecular data show that RHDV2 did not emerge following genetic evolution among previously known lagoviruses. Indeed, when comparing RHDV2 to other lagoviruses, the amino acid substitutions are distributed throughout the VP60 gene and several of them are not shared by any other known rabbit lagovirus. The emergence of RHDV2 from a non-pathogenic lagovirus has not been proven yet, but this hypothesis cannot be definitively excluded since knowledge of non-pathogenic lagoviruses remains scarce. However, a recent study based on the analysis of complete capsid gene sequences estimated an accurate evolution rate in RHDV after removing one misdated RHDV from the databases [49]. This evolution rate is higher than those previously estimated [18,19,50]. Using it, the emergence of virulence is estimated to have taken place early in the 20th century, in 1918 (95% CI: 1893–1941), i.e. well before the first occurrence of the disease in 1984. It is unlikely that such a highly pathogenic virus could have gone undetected over such a long period in a species of economic interest. Therefore, the alternative hypothesis of a species jump could be put forth to explain the emergence of RHDV, with the evolution towards virulence having occurred in a species other than the rabbit of genus *Oryctolagus*. According to this hypothesis, the emergence of RHDV2 could be due to a second lagovirus species jump from a putative original host to the rabbit, the first one being the emergence of RHDV in the 1980s. The discovery of this reservoir host species would give weight to this hypothesis and could facilitate understanding of the emergence of pathogenic lagoviruses in rabbits and perhaps also in hares.

## Competing interests

The authors declare that they have no competing interests.

## Authors’ contributions

Study development and design: GLG, SM, LC, AL, SBe, JLG. Molecular genetic studies and sequence alignments: GLG, FZ, PC, EL. Antigenic characterization: LC, AL, PC. Statistical analyses: SM. Sampling of wild and domestic rabbit populations, clinical observations and post-mortem examinations: AD, SM, AL, SBo, BLN. Experimental studies: NM, SBe, AL, LC, JLG, GL. Drafting of the paper: GLG, SM, AL, LC, SBe, JLG, PC. All the authors read and approved the final manuscript.

## Supplementary Material

Additional file 1 **Rabbit lagovirus VP60 sequences used for the phylogenetic analyses.** Sequences are ordered according to their position in the phylogenetic tree (Figure 2). The virus name corresponds to the country of origin, the name of the isolate and the year of collection (when known). For RHDV, the genetic groups are annotated according to Le Gall-Reculé et al. [16] / Kerr et al. [17] / Kinnear et al. [19]. Click here for file
