# Supplementary material for: Emergence of a new lagovirus related to Rabbit Haemorrhagic Disease Virus
Source: Vet Res. 2013 Sep 8;44(1):81. doi: 10.1186/1297-9716-44-81 (PMC3848706; doi:10.1186/1297-9716-44-81)
Supplement: Additional file 1 — Rabbit lagovirus VP60 sequences used for the phylogenetic analyses. Sequences are ordered according to their position in the phylogenetic tree (Figure 2). The virus name corresponds to the country of origin, the name of the isolate and the year of collection (when known). For RHDV, the genetic groups are annotated according to Le Gall-Reculé et al. [16] / Kerr et al. [17] / Kinnear et al. [19]. [file 1297-9716-44-81-S1.docx]

| **Rabbit lagovirus isolate**  **(country_strain/year)** | **GenBank**  **accession number** | **Phylogenetic**  **group** |
| --- | --- | --- |
| UK_Ireland18/2001 | ay928268 | G5 / 4 / B |
| UK_Ireland19/2001 | ay928269 |  |
| UK_Ramsay Island/2000 | ef363035 |  |
| UK_Ireland4/2001 | ay925210 |  |
| UK_Ireland25/2001 | ay928270 |  |
| UK_Ireland12/2001 | ay926883 |  |
| Germany_Wreizen/1996 | y15427 |  |
| Barhain/2001 | dq189077 |  |
| Germany_Jena | ef558576 |  |
| France_00-13/2000 | aj495856 |  |
| France_01-15=CYM74/2001 | fn552800 |  |
| France_05-01/2005 | am085133 |  |
| Germany_Meiningen/1993 | ef558577 | G3-G4 / 4 / B |
| Italy_BS89/1989 | x87607 |  |
| France_09-02/2009 | fr823354 |  |
| UK_Ascot/1992 | ef558575 |  |
| France_95-10/1995 | aj535094 |  |
| UK_Rainham/1993 | aj006019 |  |
| Germany_Wika/1996 | ef558574 |  |
| Germany_Frankfurt/1996 | y15424 |  |
| Germany_Frankfurt5 | ef558573 |  |
| Germany_Frankfurt12 | ef558572 |  |
| Germany_Hagenow/1990 | ef558585 |  |
| France_95-05/1995 | aj535092 |  |
| France_SD/1989 | z29514 | G1 / 2 / C |
| Spain_AST89/1989 | z49271 |  |
| Spain_MC-89/1989 | l48547 |  |
| Germany_Eisenhüttenstadt/1989 | ef558578 |  |
| France_00-08/2000 | aj319594 |  |
| Saudi Arabia/1996 | dq189078 | G2 / 3 / A |
| France_90-10/1990 | FR823355 |  |
| France_Haute Saone/1988 | u49726 |  |
| China_WX/1984 | AF402614 |  |
| Mexico_Mexico89/1989 | AF295785 |  |
| Korea_Korea-90/1990 | EU003580 |  |
| South Korea_RHF89/1989 | FJ212323 |  |
| Australia_AIN-2/2009 | GU373617 |  |
| Australia_Narrawa/2006 | EU650680 |  |
| Australia_PI-1/2009 | GU373618 |  |
| Australia_Nyngan/2005 | EU650679 |  |
| Italy_Italy-90/1990 | EU003579 |  |
| Germany_FRG/1989 | M67473 |  |
| Czech_V351/1987 | U54983 |  |
| New Zealand/1997 | AF231353 |  |
| New Zealand_NZ61/2003 | EF558580 |  |
| New Zealand_NZ54/2003 | EF558579 |  |
| USA_UT-01/2001 | eu003582 | G6 / 1 / D  (RHDVa) |
| Germany_Rossi/2002 | ef558584 |  |
| Germany_Triptis/1996 | ef558583 |  |
| China_09-SD/2009 | gu564448 |  |
| China_JX/CHA/97/1997 | dq205345 |  |
| China_TP | af453761 |  |
| South Korea_KV0801/2008 | fj212322 |  |
| France_00-Reu/2000 | aj303106 |  |
| France_99-05/1999 | aj302016 |  |
| USA_Iowa/2000 | af258618 |  |
| Germany_Dachswald/2000 | ef558582 |  |
| Japan_Hokkaido/2002/JPN/2002 | ab300693 |  |
| China_BJ/China/2009/2009 | jn165236 |  |
| China_XA/China/2010/2010 | jn165234 |  |
| China_FP/China/2009/2009 | jn165235 |  |
| USA_NY-01/2001 | eu003581 |  |
| China_whn/China/01/2005/2005 | dq069280 |  |
| China_XJ/China/2002/2002 | gu339228 |  |
| France_03-24/2003 | aj969628 |  |
| China_HYD/2005 | jf412629 |  |
| Cuba_CUB5-04/2004 | dq841708 |  |
| China_WHNRH | dq280493 |  |
| China_whn/China/02/2005/2005 | dq069281 |  |
| China_whn/China/03/2005/2005 | dq069282 |  |
| USA_IN-05/2005 | eu003578 |  |
| China_CD/China/2004 | ay523410 |  |
| Germany_Erfurt/2000 | ef558581 |  |
| China_YL | dq530363 |  |
| China_TC/China/2007/2007 | jn165233 |  |
| China_NJ-2009/2009 | hm623309 |  |
| China_WF/China/2007/2007 | fj794180 |  |
| China_SH/China/2006/2006 | fj794179 |  |
| China_NJ/China/1985/1985 | ay269825 |  |
| Russia_Manihino-09/2009 | hq917923 |  |
| Italy_RCV/1995 | x96868 | RCV-like |
| USA_MRCV/2001 | gq166866 |  |
| France_06-11/2006 | am268419 |  |
| France_10-28/2010 | HE800531 | RHDV2 |
| France_10-08/2010 | HE819400 |  |
| France_10-07/2010 | HE800530 |  |
| France_10-32/2010 | HE800532 |  |
| France_10-01/2010 | HE800529 |  |
| Italy_Ud11/2011 | JQ929052 |  |
| France_10-05/2010 | FR819781 |  |
| Australia_MIC4-9/2007 | gu373615 | RCV-A1 |
| Australia_MIC4-6/2007 | gu368907 |  |
| Australia_MIC5-8/2007 | gu368893 |  |
| Australia_MIC5-10/2007 | gu368906 |  |
| Australia_GUN1-11/2007 | gu368903 |  |
| Australia_MIC3-3/2007 | gu368892 |  |
| Australia_CAT7-1/2007 | gu368891 |  |
| Australia_CAT2-5/2007 | gu368905 |  |
| Australia_CAT2-10/2007 | gu368904 |  |
| Australia_CAT2-12/2007 | gu368889 |  |
| Australia_MIC1-5/2007 | gu373614 |  |
| Australia_MIC-07/2007 | eu871528 |  |
| Australia_BUR1-1/2007 | gu368888 |  |
| Australia_WAU-1/2009 | gu368901 |  |
| Australia_CAT3-4/2007 | gu368890 |  |
| Australia_BM-49/2009 | gu368919 |  |
| Australia_BM-40/2009 | gu368916 |  |
| Australia_BM-58/2009 | gu368918 |  |
| Australia_BM-41/2009 | gu368917 |  |
| Australia_BEN-26/2009 | gu368915 |  |
| Australia_BEN-16/2009 | gu368914 |  |
| Australia_BEN-12/2009 | gu368913 |  |
| Australia_BEN-35/2009 | gu368902 |  |
| Australia_V-8/2007 | gu368900 |  |
| Australia_V-5/2007 | gu368899 |  |
| Australia_OC-13/2007 | gu368894 |  |
| Australia_OC-20/2007 | gu368908 |  |
| Australia_OC-26/2007 | gu368896 |  |
| Australia_OC-36/2007 | gu368897 |  |
| Australia_V-11/2007 | gu373616 |  |
| Australia_OC-7/2007 | gu368898 |  |
| Australia_OC-15/2007 | gu368895 |  |
| Australia_OC-39/2007 | gu368911 |  |
| Australia_OC-33/2007 | gu368910 |  |
| Australia_OC-40/2007 | gu368912 |  |
| Australia_OC-21/2007 | gu368909 |  |
